# Evaluation of the effects of olodaterol on exercise endurance in patients with chronic obstructive pulmonary disease: results from two 6-week crossover studies

**DOI:** 10.1186/s12931-016-0389-5

**Published:** 2016-07-06

**Authors:** François Maltais, Anne-Marie Kirsten, Alan Hamilton, Dorothy De Sousa, Florian Voß, Marc Decramer

**Affiliations:** Centre de Recherche, Institut Universitaire de Cardiologie et de Pneumologie de Québec, 2725 Chemin Sainte Foy, Québec, G1V 4G5 Canada; Pulmonary Research Institute at Lung Clinic Grosshansdorf, Airway Research Center North, Member of the German Center for Lung Research, Grosshansdorf, Germany; Boehringer Ingelheim, Burlington, ON Canada; Boehringer Ingelheim Pharma GmbH & Co. KG, Ingelheim, Germany; Department of Pulmonology, University Hospitals Leuven, Leuven, Belgium

**Keywords:** Olodaterol, Bronchodilator, Long-acting β_2_-agonist, COPD

## Abstract

**Background:**

Two replicate, double-blind, placebo-controlled, 6-week crossover studies assessed the effect of the once-daily long-acting β_2_-agonist olodaterol 5 μg and 10 μg on constant work-rate cycle endurance in patients with moderate to very severe chronic obstructive pulmonary disease.

**Methods:**

Patients received placebo, olodaterol 5 μg once daily (QD) and olodaterol 10 μg QD in a randomised order for 6 weeks each, with a 2-week washout period in between. The primary end point was change in endurance time during constant work-rate cycle ergometry to symptom limitation at 75 % maximal work capacity after 6 weeks of treatment (2 h post-dose), based on log_10_-transformed data. Key secondary end points were inspiratory capacity at isotime and intensity of breathing discomfort at isotime.

**Results:**

151 and 157 patients were randomised and treated in Studies 1222.37 and 1222.38, respectively, with 147 and 154 being included in the full analysis sets. Mean endurance time at week 6 was increased compared to placebo by 14.0 % (Study 1222.37; *p* < 0.001) and 11.8 % (Study 1222.38; *p* < 0.01) with olodaterol 5 μg, and by 13.8 % (Study 1222.37; *p* < 0.001) and 10.5 % (Study 1222.38; *p* < 0.01) with olodaterol 10 μg. Inspiratory capacity at isotime increased with olodaterol 5 μg (Study 1222.37, 0.182 L, *p* < 0.0001; Study 1222.38, 0.084 L, *p* < 0.05) and 10 μg (Study 1222.37, 0.174 L; Study 1222.38, 0.166 L; both studies, *p* < 0.0001), and breathing discomfort was significantly reduced in Study 1222.37 (olodaterol 5 μg, 0.77 Borg units, *p* < 0.001; olodaterol 10 μg, 0.63 Borg units, *p* < 0.01) but not Study 1222.38.

**Conclusions:**

These studies provide further characterisation of the efficacy of olodaterol, showing that improvements in airflow (forced expiratory volume in 1 s) are associated with increases in inspiratory capacity and improvements in exercise endurance time.

**Trial registrations:**

NCT01040130 (1222.37) and NCT01040793 (1222.38).

**Electronic supplementary material:**

The online version of this article (doi:10.1186/s12931-016-0389-5) contains supplementary material, which is available to authorized users.

## Background

Expiratory flow limitation is a hallmark of chronic obstructive pulmonary disease (COPD) [1]. During periods of increased ventilatory demand, expiratory flow limitation results in dynamic hyperinflation and is associated with significant breathing discomfort [2]. Dynamic lung hyperinflation is seen in the majority of patients with COPD during cycling [2–4] and may also be present in daily activities such as walking [5]. Hyperinflation limits exercise tolerance, which, in turn, reduces patients’ quality of life [6] and, potentially, survival [7]. As such, improving exercise tolerance is a key therapeutic goal in COPD [8].

Long-acting β_2_-agonists (LABAs) and long-acting muscarinic antagonists (LAMAs) are well established as maintenance therapies for moderate to very severe COPD [8, 9]. Improvements in airflow limitation with both LAMAs and LABAs are associated with increases in inspiratory capacity (IC) at rest and during exercise in patients with COPD (a marker of reduced lung hyperinflation), with resultant reductions in breathing discomfort and improvements in exercise endurance time [2, 10–17].

Olodaterol is a novel LABA with a high affinity for, and almost full intrinsic activity at, β_2_ receptors, and a low affinity and partial agonist activity at β_1_ receptors [18, 19]. Its duration of action is ≥24 h, allowing for once-daily (QD) dosing [20]. Pivotal Phase III trials have established the long-term efficacy of olodaterol 5 μg and 10 μg QD with respect to lung function (forced expiratory volume in 1 s [FEV_1_]) [21–24].

Studies 1222.37 and 1222.38 were designed to test the hypothesis that olodaterol reduces airflow limitation during tidal breathing, reducing hyperinflation and breathing discomfort experienced during exercise, with consequent improvement in symptom-limited cycling exercise endurance.

The aim of these two replicate, 6-week, placebo-controlled, crossover studies was thus to assess the effects of olodaterol 5 μg and 10 μg QD, via the Respimat® inhaler, on constant work-rate cycling exercise endurance in patients with moderate to very severe COPD.

## Methods

### Patients

Patients with moderate to very severe COPD (Global initiative for chronic Obstructive Lung Disease [GOLD] 2–4) were included in the studies if they met the following inclusion criteria: persistent airway obstruction with post-bronchodilator FEV_1_ <80 % of predicted normal and post-bronchodilator FEV_1_/forced vital capacity [FVC] <70 %; aged 40–75 years; and with a smoking history of >10 pack-years. At variance with many COPD exercise endurance trials [2, 12–15, 17], the presence of static lung hyperinflation (i.e., increased functional residual capacity [FRC]) was not an entry requirement, since the intention of the study was to evaluate the effects of olodaterol on exercise endurance in a broad patient population. Key exclusion criteria included: a significant disease other than COPD that could influence patients’ safety during the study; history of asthma; myocardial infarction in the previous year; unstable or life-threatening cardiac arrhythmia; or hospitalisation due to heart failure in the previous year. In addition, patients were not eligible to take part if their exercise performance was limited for a reason other than fatigue or dyspnoea, such as arthritis, angina pectoris, claudication or morbid obesity, or if they had any contraindications to exercise as outlined by the European Respiratory Society Task Force on clinical exercise testing [25]. Patients with a cycling endurance time of ≥25 min at pre-randomisation evaluation were also excluded.

### Study design

**Fig. 1:**
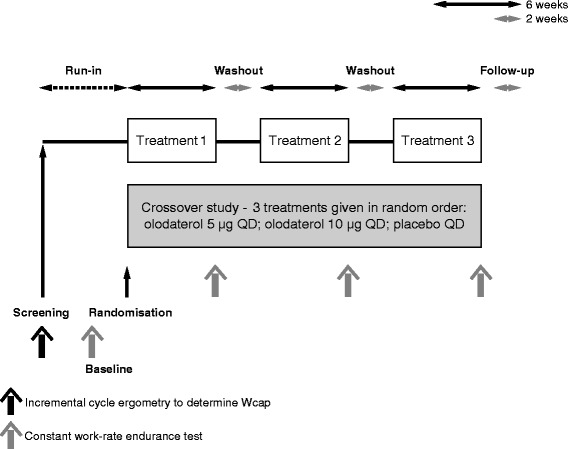
Trial design for Studies 1222.37 and 1222.38. *QD* once daily, *Wcap* maximum work capacity

Studies 1222.37 (NCT01040130) and 1222.38 (NCT01040793) were replicate, multicentre, multinational, randomised, double-blind, placebo-controlled, three-way crossover trials (see Fig. 1 for a schematic of the study design). Following an initial screening visit, there was a 2-week baseline period. During the baseline period and for the rest of the trial, patients were permitted to continue with short-acting muscarinic antagonists (at least 8-h washout required prior to clinic visits), inhaled corticosteroids and xanthines, and rescue short-acting β_2_-agonists (open-label salbutamol), but not LAMAs or LABAs.

Patients received each of the following treatments for 6 weeks in a randomised order: olodaterol 5 μg QD, olodaterol 10 μg QD and placebo QD. Olodaterol was administered as two actuations of the Respimat® inhaler. Between treatment periods, there was a 2-week washout period where patients continued with their permitted therapy. Clinic visits were scheduled on days 1 and 43 of each treatment period, with a follow-up visit 2 weeks after the last treatment period. In the case of early discontinuation, a follow-up visit was completed 2 weeks after the final dose of study medication.

The studies were carried out in accordance with the principles of the Declaration of Helsinki and the International Conference on Harmonisation Harmonised Tripartite Guideline for Good Clinical Practice, and written, informed consent was obtained from each patient.

### Exercise testing

At the initial screening visit, maximum work capacity (Wcap) was determined for each patient during incremental cycle ergometry conducted as described by O’Donnell and Webb [26]. Prior to randomisation, patients performed a ‘training’ constant work-rate cycle endurance test to symptom limitation at 75 % of Wcap and, ≥2 days later, performed a second constant work-rate cycle endurance test to determine pre-treatment baseline endurance time. Constant work-rate cycle endurance tests at 75 % Wcap were repeated on day 43 of each treatment period at 2 h (+ ≤15 min) after inhalation of the study medication. To limit the number of exercise tests performed by patients, the pre-randomisation test was used as baseline for all treatment comparisons. Intensity of breathing discomfort using the Borg category-ratio scale was recorded and IC was measured at rest, at 2-min intervals during exercise and at the end of exercise, as previously described [13]. Heart rate, blood pressure and electrocardiogram measurements were also recorded during exercise. After completing each exercise test, patients indicated the reason for stopping exercise using a simple questionnaire (due to leg and/or breathing discomfort, chest pain or other reason).

### Pulmonary function testing

Spirometry (FEV_1_, FVC and peak expiratory flow) was performed at screening and on days 1 and 43 of each treatment period, 30 min pre-dose (trough measurement) and 1 h post-dose.

Body plethysmography was performed on days 1 and 43 of each treatment period 30 min pre-dose (trough measurement) and 1 h post-dose (prior to spirometry), according to the methods and calibration described by Coates et al. [27] to determine FRC and IC, with total lung capacity calculated as mean FRC + largest IC of three plethysmographic measurements.

### Outcome measures

The primary end point was log_10_-transformed endurance time during constant work-rate cycle ergometry to symptom limitation at 75 % Wcap after 6 weeks of treatment. Key secondary end points were IC and intensity of breathing discomfort at isotime. Isotime was defined for each patient as the furthest exercise time that they reached in all of the constant work-rate tests (baseline and all treatment periods), i.e., their shortest ever endurance time.

In addition, two *post hoc* subgroup analyses were performed using combined data from Studies 1222.37 and 1222.38 to investigate exercise time in patients with static hyperinflation (FRC ≥120 % predicted) and with static and/or dynamic hyperinflation (defined as IC at rest – IC end exercise >100 mL [28]). Additional *post hoc* analyses (using data from the individual studies) were conducted to compare exercise time in GOLD 2 patients to GOLD 3 or 4 patients.

### Statistical analyses

For the primary end point, adjusted means of endurance time on a log_10_ scale were tested using a mixed model for repeated measures, based on previous studies showing that endurance time has a log normal distribution [4]. The model included treatment and period as fixed effects and patient as a random effect. Log_10_-transformed study baseline endurance time was added as a covariate.

Based on a predicted standard deviation of within-subject treatment difference for endurance time on a log_10_ scale of ~0.181 s, with a Type I error rate of 0.05 (two-sided), 102 patients were required to detect a difference in endurance time of 15 %. Allowing for possibly higher standard deviation and patient dropout, 150 patients needed to be randomised in each study.

The primary analysis was conducted on the full analysis set, which included all patients with baseline and any evaluable post-dose endurance time data. A sensitivity analysis for the primary end point was performed based on a per-protocol set, which included patients with no significant protocol violations. No imputation was made for missing endurance time values.

Secondary analyses used the mixed model for repeated measures described above for all continuous variables but without using a log_10_ scale and with non-transformed study baseline as a covariate. To calculate the IC and Borg scale values at isotime when no value was available at that exact time point, interpolation was to be used if a value was available afterwards. Missing IC or Borg scale values were imputed using last observation carried forward.

The primary and key secondary end points were included in a hierarchical testing strategy, shown in Additional file 1: Figure S1, with each test considered confirmatory only if all of the previous tests were successful. There was no alpha protection for multiple testing for the additional secondary end points.

## Results

### Patient disposition and baseline characteristics

A total of 151 patients (from 19 sites in five countries) and 157 patients (from 19 sites in five countries) were randomised into the treatment phases in Study 1222.37 and Study 1222.38, respectively (Additional file 1: Figures S2a and b). Overall, 147 (Study 1222.37) and 154 (Study 1222.38) patients were included in the full analysis set for primary analyses. At least 95 % (Study 1222.37) and 94 % (Study 1222.38) of patients in each treatment arm completed the full 6 weeks; patients who discontinued a treatment period were permitted to continue to the next treatment (Additional file 1: Figures S2a and b).

Baseline patient characteristics are shown in Table 1. Patients in Study 1222.38 had a higher baseline mean pre-bronchodilator FEV_1_ and lower mean change from pre- to post-bronchodilator FEV_1_ than in Study 1222.37 (Table 1). While there was no specific requirement for the presence of static lung hyperinflation at study entry, 108 (71.5 %) patients in Study 1222.37 and 116 (73.9 %) patients in Study 1222.38 did exhibit resting lung hyperinflation (FRC ≥120 % predicted normal at baseline) (Table 1). Relevant parameters during the baseline constant work-rate cycle test are shown in Table 2. An overview of pulmonary medication use prior to study enrolment is presented in Additional file 1: Table S1.

**Table 1:** Baseline demographics and patient characteristics (treated set^a^)

	Study 1222.37	Study 1222.38
	(*n* = 151)	(*n* = 157)
Male, n (%)	116 (76.8)	116 (73.9)
Mean (SD) age, years	60.6 (7.7)	60.6 (7.7)
Smoking status, n (%)		
Ex-smoker Current smoker	84 (55.6) 67 (44.4)	92 (58.6) 65 (41.4)
Mean (SD) smoking history, pack-years	45.3 (22.5)	50.0 (29.0)
Mean (SD) pre-bronchodilator at screening		
FEV_1_, L FEV_1_ % predicted	1.46 (0.54) 48.5 (14.5)	1.56 (0.53) 51.6 (14.2)
Mean (SD) post-bronchodilator at screening		
FEV_1_, L Change from pre- to post-bronchodilator	1.66 (0.55)	1.70 (0.53)
FEV_1_, L	0.19 (0.18)	0.14 (0.19)
FEV_1_ % predicted	55.1 (14.4)	56.1 (13.1)
Change from pre- to post-bronchodilator		
FEV_1_, %	15.7 (14.7)	10.9 (14.3)
GOLD, n (%)		
1 (≥80 %) 2 (50– <80 %) 3 (30– <50 %) 4 (<30 %)	0 (0.0) 100 (66.2) 43 (28.5) 8 (5.3)	1 (0.6)^b^ 111 (70.7) 41 (26.1) 4 (2.5)
Patients with FRC ≥120 % predicted normal, n (%)	108 (71.5)	116 (73.9)

^a^4 patients in Study 1222.37 and 3 in Study 1222.38 were included in the treated set but not the full analysis set because they did not have baseline or primary end point data; ^b^1 patient with a predicted FEV_1_ of 80.4 %. This was classed as a protocol violation; the patient was included in the full analysis set but was excluded from the per protocol set

*SD* standard deviation, *FEV*_*1*_ forced expiratory volume in 1 s, *GOLD* Global initiative for chronic Obstructive Lung Disease, *FRC* functional residual capacity

**Table 2:** Baseline exercise parameters (full analysis set)

	Study 1222.37	Study 1222.38
	(*n* = 147)	(*n* = 154)
Geometric mean (SE) endurance time, s	414.2 (18.5)	373.9 (13.5)
Arithmetic mean (SE) endurance time, s	478.2 (21.9)	415.5 (17.4)
Mean (SE) IC, L		
Pre-exercise Isotime End-exercise	2.29 (0.06) 2.04 (0.07) 2.00 (0.06)	2.37 (0.06) 2.14 (0.06) 2.13 (0.06)
Mean (SE) breathing discomfort, Borg units		
Pre-exercise Isotime End-exercise	0.32 (0.05) 5.27 (0.21) 7.20 (0.20)	0.31 (0.05) 5.53 (0.19) 7.51 (0.18)
Mean (SE) leg discomfort, Borg units		
Pre-exercise Isotime End-exercise	0.26 (0.07) 5.27 (0.22) 7.05 (0.23)	0.18 (0.04) 4.42 (0.22) 6.01 (0.24)
Locus of symptom limitation, n (%)		
Breathing discomfort Leg discomfort Breathing and leg discomfort None	52 (35.4) 40 (27.2) 52 (35.4) 3 (2.0)	81 (52.6) 26 (16.9) 42 (27.3) 5 (3.2)

*SE* standard error, *IC* inspiratory capacity

### Efficacy

#### Endurance time

The assumption of a log normal distribution was confirmed in both studies, with clear differences between the arithmetic mean and median endurance times at baseline and after 6 weeks of treatment in all treatment arms (Additional file 1: Table S2, Figure S3); thus the validity of the pre-specified primary analysis based on log_10_-transformed endurance time was confirmed.

**Fig. 2:**
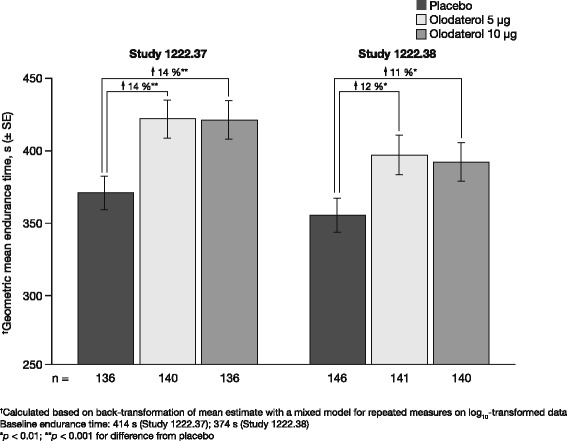
Adjusted geometric mean symptom-limited endurance time after 6 weeks in Studies 1222.37 and 1222.38 (full analysis set). *SE* standard error

Log_10_-transformed mean endurance time at 6 weeks significantly increased by 11.8–14.0 % with olodaterol 5 μg and by 10.5–13.8 % with olodaterol 10 μg when compared to placebo (Fig. 2). Arithmetic and geometric mean endurance times are shown in Additional file 1: Table S2.

There were no significant differences in endurance time between olodaterol 5 μg and 10 μg for the primary end point in either study. The results of the per-protocol set analysis were consistent with the primary analysis.

Changes in endurance time in patients who were GOLD 2 and patients who were GOLD 3 or 4 are shown in Additional file 1: Table S3. There were generally slightly higher increases in endurance time with olodaterol in GOLD 3/4 patients than in GOLD 2 patients.

In patients from Studies 1222.37 and 1222.38 with static hyperinflation at baseline (FRC ≥120 % predicted normal; *n* = 224), log_10_-transformed mean endurance time at 6 weeks significantly increased by 13.2 % (*p* < 0.0001) and 12.2 % (*p* = 0.0002) with olodaterol 5 μg and 10 μg, respectively, from a geometric mean baseline endurance time of 376.4 s. In a subgroup of patients from both studies with static and/or dynamic hyperinflation (defined as IC at rest – IC end exercise >100 mL [28]; *n* = 274), log_10_ mean endurance time increased from geometric mean baseline of 388.2 s by 13.3 % (*p* < 0.0001) and 12.1 % (*p* < 0.0001) with olodaterol 5 μg and 10 μg, respectively. These improvements were of similar magnitude to those observed in the whole population of patients.

#### IC and breathing discomfort

**Fig. 3:**
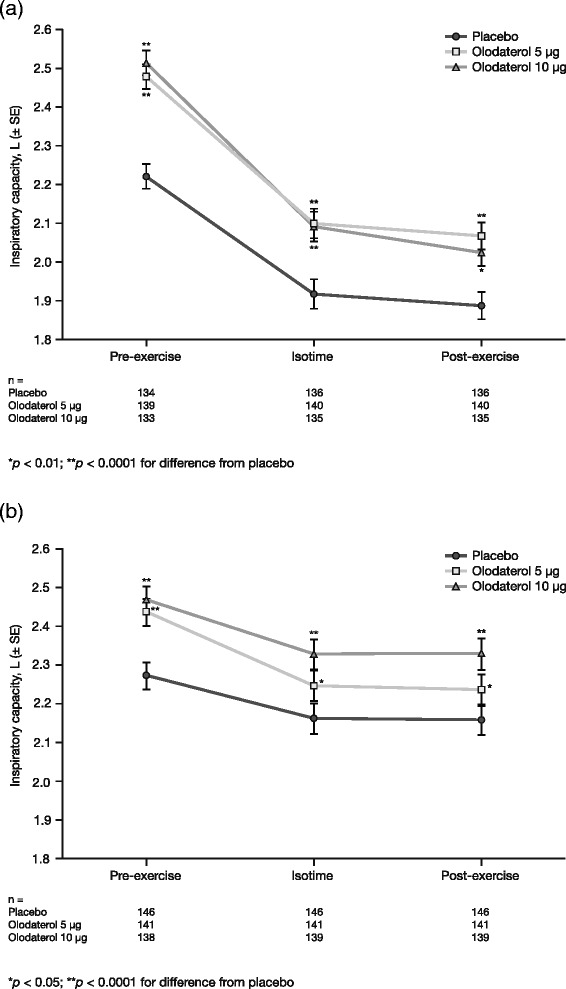
Adjusted mean inspiratory capacity after 6 weeks in (**a**) Study 1222.37 and (**b**) Study 1222.38 (full analysis set). *SE* standard error

In both studies, IC increased with olodaterol 5 μg and 10 μg at rest (prior to exercise), at isotime and at end-exercise (Fig. 3). There was a statistically significant increase in IC at isotime compared to placebo with olodaterol 5 μg (0.182 L; *p* < 0.0001) and 10 μg (0.174 L; *p* < 0.0001) in Study 1222.37, and with olodaterol 5 μg (0.084 L; *p* < 0.05) and 10 μg (0.166 L; *p* < 0.0001) in Study 1222.38 (Fig. 3).

There was a significant reduction in the intensity of breathing discomfort at isotime compared to placebo in Study 1222.37 of 0.77 Borg units with olodaterol 5 μg (*p* < 0.001) and 0.63 Borg units with olodaterol 10 μg (*p* < 0.01); there were no significant differences in Study 1222.38 (Table 3).

**Table 3:** Adjusted mean breathing discomfort measured using the Borg category-ratio scale at isotime after 6 weeks (full analysis set)

		Isotime		
Treatment	n	Mean time, mm:ss^a^	Borg (SE)	*p* value versus placebo
Study 1222.37				
Baseline			5.27 (0.21)	
Placebo	136	05:58	5.87 (0.19)	
Olodaterol 5 μg	140	06:02	5.10 (0.18)	0.0007
Olodaterol 10 μg	136	06:05	5.24 (0.19)	0.0051
Study 1222.38				
Baseline			5.53 (0.19)	
Placebo	146	05:29	5.59 (0.18)	
Olodaterol 5 μg	141	05:25	5.25 (0.18)	0.1176
Olodaterol 10 μg	140	05:22	5.52 (0.18)	0.7591

^a^Due to differences in the number of patients between treatment groups, slight differences in mean time are observed

*SE* standard error

#### Spirometry and body plethysmography

In both studies, FEV_1_ and FVC significantly improved at trough (30 min pre-dose) and at 1 h post-dose with olodaterol 5 μg and 10 μg compared to placebo (Table 4). Peak expiratory flow data are shown in Additional file 1: Table S4.

**Table 4:** Adjusted mean FEV_1_ and FVC outcomes at 6 weeks (full analysis set)

		FEV_1_			FVC		
Treatment	Planned time (min)	Mean (SE), L	Mean (SE) difference from placebo	*p* value	Mean (SE), L	Mean (SE) difference from placebo	*p* value
Study 1222.37							
Placebo	−0.30 (pre-dose/trough)	1.475 (0.017)^a^			3.212 (0.037)^b^		
Olodaterol 5 μg		1.564 (0.017)^c^	0.089 (0.017)	<0.0001	3.319 (0.037)^a^	0.107 (0.031)	0.0006
Olodaterol 10 μg		1.576 (0.017)^c^	0.101 (0.017)	<0.0001	3.310 (0.037)^a^	0.098 (0.031)	0.0017
Placebo	60 (post-dose)	1.473 (0.019)^a^			3.187 (0.034)^b^		
Olodaterol 5 μg		1.698 (0.019)^c^	0.224 (0.017)	<0.0001	3.471 (0.034)^a^	0.285 (0.029)	<0.0001
Olodaterol 10 μg		1.699 (0.019)^c^	0.226 (0.017)	<0.0001	3.477 (0.034)^a^	0.290 (0.029)	<0.0001
Study 1222.38							
Placebo	−0.30 (pre-dose/trough)	1.520 (0.024)^d^			3.103 (0.039)^d^		
Olodaterol 5 μg		1.630 (0.025)^e^	0.110 (0.019)	<0.0001	3.222 (0.040)^e^	0.119 (0.036)	0.0013
Olodaterol 10 μg		1.630 (0.025)^f^	0.110 (0.019)	<0.0001	3.222 (0.040)^f^	0.119 (0.037)	0.0013
Placebo	60 (post-dose)	1.577 (0.026)^d^			3.144 (0.039)^d^		
Olodaterol 5 μg		1.768 (0.026)^e^	0.192 (0.021)	<0.0001	3.409 (0.040)^e^	0.265 (0.035)	<0.0001
Olodaterol 10 μg		1.771 (0.026)^f^	0.195 (0.021)	<0.0001	3.425 (0.040)^f^	0.281 (0.035)	<0.0001

^a^ *n* = 136; ^b^ *n* = 135; ^c^ *n* = 137; ^d^ *n* = 146; ^e^ *n* = 143; ^f^ *n* = 139

Common baseline means (SE): Study 1222.37, FEV_1_, 1.478 (0.043); FVC, 3.221 (0.073). Study 1222.38, FEV_1_, 1.553 (0.043); FVC, 3.160 (0.072)

*SE* standard error, *FEV*_*1*_ forced expiratory volume in 1 s, *FVC* forced vital capacity

Both studies showed statistically significant increases in IC measured using body plethysmography for olodaterol 5 μg and 10 μg compared to placebo at 30 min pre-dose and 1 h post-dose, with corresponding decreases in FRC (Table 5). The results of IC at 1 h post-dose using body plethysmography (Table 5) were similar to the results of IC 2 h post-dose measured using spirometry immediately prior to exercise (Fig. 3).

**Table 5:** Adjusted mean body plethysmography outcomes at 6 weeks: IC and FRC (full analysis set)

			IC			FRC		
Treatment	Planned time (min)	n^a^	Mean (SE) IC, L	Mean (SE) difference from placebo, L	*p* value	Mean (SE) FRC, L	Mean (SE) difference from placebo, L	*p* value
Study 1222.37								
Placebo	−30 (pre-dose/trough)	134-135	2.170 (0.040)			4.977 (0.062)		
Olodaterol 5 μg		139-140	2.289 (0.040)	0.119 (0.034)	0.0005	4.855 (0.061)	−0.122 (0.069)	0.0784
Olodaterol 10 μg		134	2.262 (0.040)	0.092 (0.034)	0.0073	4.862 (0.062)	−0.115 (0.070)	0.1013
Placebo	60 (post-dose)	134-135	2.221 (0.040)			4.950 (0.064)		
Olodaterol 5 μg		139-140	2.427 (0.040)	0.206 (0.035)	<0.0001	4.740 (0.063)	−0.210 (0.066)	0.0015
Olodaterol 10 μg		134	2.437 (0.040)	0.216 (0.036)	<0.0001	4.577 (0.064)	−0.373 (0.066)	<0.0001
Study 1222.38								
Placebo	−30 (pre-dose/trough)	147	2.463 (0.041)			4.842 (0.062)		
Olodaterol 5 μg		145-146	2.613 (0.041)	0.150 (0.040)	0.0002	4.757 (0.063)	−0.086 (0.053)	0.1048
Olodaterol 10 μg		141-142	2.618 (0.042)	0.154 (0.040)	0.0001	4.723 (0.063)	−0.120 (0.053)	0.0246
Placebo	60 (post-dose)	147	2.493 (0.040)			4.770 (0.065)		
Olodaterol 5 μg		145-146	2.725 (0.040)	0.232 (0.036)	<0.0001	4.557 (0.065)	−0.213 (0.053)	<0.0001
Olodaterol 10 μg		141-142	2.696 (0.040)	0.203 (0.036)	<0.0001	4.583 (0.065)	−0.187 (0.054)	0.0005

^a^N numbers for IC and FRC

*IC* inspiratory capacity, *FRC* forced residual capacity, *SE* standard error

### Safety and tolerability

#### Adverse events

Across both studies, incidences of adverse events, serious adverse events and adverse events leading to discontinuation with olodaterol 5 μg and 10 μg were similar to placebo (Table 6). The most common adverse event was COPD exacerbation, occurring in 6.7–7.0 %, 6.0–7.5 % and 2.7–4.9 % of patients with placebo, olodaterol 5 μg and olodaterol 10 μg, respectively, in both studies. Three patients in each treatment group in Study 1222.37 and three patients in the placebo group, eight with olodaterol 5 μg and three with olodaterol 10 μg in Study 1222.38 had serious adverse events. None of the serious adverse events in Study 1222.37 and one in Study 1222.38 (atrial fibrillation in a patient taking olodaterol 5 μg) were considered by the investigator to be related to the study drug.

**Table 6:** Common adverse events^a^ (treated set^b^)

	Study 1222.37, n (%)			Study 1222.38, n (%)		
	Placebo	Olodaterol 5 μg	Olodaterol 10 μg	Placebo	Olodaterol 5 μg	Olodaterol 10 μg
	(*n* = 143)	(*n* = 147)	(*n* = 143)	(*n* = 149)	(*n* = 150)	(*n* = 147)
Any adverse event	38 (26.6)	49 (33.3)	42 (29.4)	34 (22.8)	42 (28.0)	31 (21.1)
Serious adverse event	3 (2.1)	3 (2.0)	3 (2.1)	3 (2.0)	8 (5.3)	3 (2.0)
Adverse event leading to discontinuation	4 (2.8)	4 (2.7)	2 (1.4)	1 (0.7)	2 (1.3)	0 (0.0)
Most common adverse events						
COPD exacerbation Nasopharyngitis Insomnia Headache Dyspnoea Cough Oropharyngeal pain	10 (7.0) 4 (2.8) 0 (0.0) 1 (0.7) 3 (2.1) 2 (1.4) 1 (0.7)	11 (7.5) 3 (2.0) 2 (1.4) 4 (2.7) 4 (2.7) 1 (0.7) 0 (0.0)	7 (4.9) 1 (0.7) 3 (2.1) 2 (1.4) 2 (1.4) 3 (2.1) 3 (2.1)	10 (6.7) 3 (2.0) 0 (0.0) 0 (0.0) 0 (0.0) 2 (1.3) 0 (0.0)	9 (6.0) 2 (1.3) 0 (0.0) 2 (1.3) 1 (0.7) 1 (0.7) 0 (0.0)	4 (2.7) 7 (4.8) 0 (0.0) 2 (1.4) 2 (1.4) 0 (0.0) 0 (0.0)

^a^With incidence ≥2 %; ^b^4 patients in Study 1222.37 and 3 in Study 1222.38 were included in the treated set but not the full analysis set because they did not have baseline or primary end point data

*COPD* chronic obstructive pulmonary disease

There were no clinically relevant changes in laboratory parameters, vital signs or electrocardiogram in either study. There were no deaths in Study 1222.37 and one death of unknown cause after 5 days of treatment in the olodaterol 10 μg group in Study 1222.38.

## Discussion

Results from these two replicate trials confirm that treatment with olodaterol improves exercise endurance time during constant work-rate cycle ergometry in patients with COPD. The improvements in endurance time were observed in patients with a broad range of disease characteristics, including GOLD 2 patients with moderate disease (69 % of the total patient population) and patients with and without static lung hyperinflation (89 % of the total patient population), supporting the application of our results to a larger COPD population. There were no differences in endurance time between patients receiving olodaterol 5 μg and 10 μg, and both doses were well tolerated. The improved endurance time with olodaterol was associated with an increase in IC that was present at rest (prior to exercise) and was maintained during exercise, which has also been observed with other bronchodilators [13].

Since early exercise studies of tiotropium in patients with COPD [13, 15], constant work-rate exercise protocols to symptom limitation, with serial measurements of IC and breathing discomfort, have frequently been used to investigate the effects of long-acting bronchodilators on lung hyperinflation, exertional dyspnoea and symptom-limited exercise capacity [12, 14, 15, 17, 29–31] (NCT01533922 and NCT01533935; manuscript in preparation). The consistent demonstration of a significant relationship between IC and endurance time [13] has led to an increased understanding of the mechanistic relationship between the primary effects of bronchodilators in improving expiratory flow, and the consequent reductions in lung hyperinflation and exertional breathing discomfort, which in turn lead to increases in the time to reach intolerable symptom intensity during exercise.

An important methodological consideration in relation to the current trials is that statistical analyses were performed on log_10_-transformed endurance time data, unlike previous trials that have assessed cycling endurance time in COPD. Although these analyses are justified by the fact that endurance time during constant work-rate exercise is asymmetrically distributed around the mean, with a significant skew towards long exercise endurance times (as has been previously reported [4] and confirmed in the present trials), this approach complicates comparisons with previous trials. For the purpose of facilitating comparisons across studies, we also report the arithmetic mean treatment effects of olodaterol (Additional file 1: Table S2). The arithmetic differences in endurance time for olodaterol, compared to placebo, range from 40 to 64 s. Although these may appear to be at the lower end of what is typically reported with other long-acting bronchodilators, including tiotropium, glycopyrronium bromide, aclidinium bromide, salmeterol and indacaterol [12, 14, 15, 17, 29, 30], they are within the estimated range for a clinically meaningful difference, compared to placebo, of 46–105 s for endurance time, as proposed by the European Respiratory Society task force on outcomes in COPD [32].

Caution should be exercised when comparing increases in endurance time across studies due to a number of confounding factors, including differences in patient characteristics and phenotypes. Variation in the improvement in expiratory flow following bronchodilation from one study population to another may contribute to the observed differences in effect size between studies. In light of this, an important methodological difference between our studies and previous exercise trials was that the presence of static lung hyperinflation was not a prerequisite for participation in our trials. Also at variance with previous trials [13, 15], patients with post-bronchodilator FEV_1_ ≤80 % predicted were permitted in our trials, including a larger proportion of GOLD 2 patients (69 % of the total patient population). Although olodaterol also enhanced exercise capacity in these patients, the inclusion of patients with milder COPD may have mitigated the observed effect sizes of the study medication on exercise capacity to some extent.

The multifactorial nature of exercise limitation in patients with COPD may also contribute to the variability in exercise response to bronchodilators from one study to another. This phenomenon is illustrated by the results of replicate studies with tiotropium [13, 15], in which similar lung-function improvements resulted in notable differences in the magnitude of improvement in endurance time, varying from 21 % in the first study to 40 % in the second study.

An unexpected finding in the present studies was the reduction in endurance time in the placebo group after 6 weeks of treatment, compared to pre-randomisation baseline, which has not been observed in previous studies. Whether this was a reflection of a time-dependent decrease in exercise capacity over the 23-week observation period or a deterioration related to the lack of optimal bronchodilation in the placebo group is difficult to ascertain because only a single, pre-randomisation baseline exercise test was included to limit the burden on study participants. However, the concomitant reduction in IC that was also observed prior to exercise in the placebo group would argue for the latter explanation. A reduction in post-treatment exercise endurance, compared to pre-randomisation baseline, was also observed in the placebo groups of two recently completed, replicate, 6-week, crossover trials of similar design (NCT01533922 and NCT01533935; manuscript in preparation), while in a 12-week, parallel-group, exercise study, exercise endurance time in the placebo group was reduced after 6 weeks, but increased after 12 weeks, compared to baseline (NCT01525615; manuscript in preparation).

## Conclusions

These two studies demonstrated that olodaterol 5 μg and 10 μg both improved exercise endurance time after 6 weeks of treatment compared to placebo, likely as a consequence of reductions in hyperinflation prior to exercise, which were maintained during exercise.

## Abbreviations

COPD, chronic obstructive pulmonary disease; FEV_1_, forced expiratory volume in 1 s; FRC, functional residual capacity; FVC, forced vital capacity; GOLD, Global initiative for chronic Obstructive Lung Disease; IC, inspiratory capacity; LABA, long-acting β_2_-agonist; LAMA, long-acting muscarinic antagonist; QD, once daily; Wcap, maximum work capacity

## Additional file

Additional file 1:
**Table S1.** Pulmonary medication use prior to enrolment (treated set). **Table S2.** Arithmetic mean, geometric mean and median endurance times after 6 weeks (full analysis set). **Table S3.** Geometric mean (SE) endurance time after 6 weeks by GOLD (full analysis set). **Table S4.** Peak expiratory flow outcomes at 6 weeks (full analysis set). **Figure S1.** Hierarchical testing order: each test was considered confirmatory only if all of the previous tests were positive. **Figure S2.** Participant flow in (a) Study 1222.37 and (b) Study 1222.38. **Figure S3.** Exercise endurance time after 6 weeks for (a) Study 1222.37 and (c) Study 1222.38. Log_10_-transformation of exercise endurance time after 6 weeks for (b) Study 1222.37 and (d) Study 1222.38 (full analysis set). (DOCX 631 kb)
