# Supplementary material for: Evaluation of the effects of olodaterol on exercise endurance in patients with chronic obstructive pulmonary disease: results from two 6-week crossover studies
Source: Respir Res. 2016 Jul 6;17:77. doi: 10.1186/s12931-016-0389-5 (PMC4936013; doi:10.1186/s12931-016-0389-5)

**Table S1** Pulmonary medication use at baseline (treated set)

| Pulmonary medication class, n (%) | Study | |
| --- | --- | --- |
|  | 1222.37 (n = 151) | 1222.38 (n = 157) |
| LAMA monotherapy | 30 (19.9) | 30 (19.1) |
| LABA monotherapy | 8 (5.3) | 3 (1.9) |
| LABA/ICS and no LAMA | 11 (7.3) | 23 (14.6) |
| LAMA/ICS and no LABA | 1 (0.7) | 1 (0.6) |
| LAMA/LABA and no ICS | 15 (9.9) | 4 (2.5) |
| LAMA/LABA/ICS triple therapy | 39 (25.8) | 23 (14.6) |

LAMA: long-acting muscarinic antagonist; LABA: long-acting β_2_-agonist; ICS: inhaled corticosteroid

**Table S2** Arithmetic mean, geometric mean and median endurance times after 6 weeks (full analysis set)

|  | Study 1222.37 | | | Study 1222.38 | | |
| --- | --- | --- | --- | --- | --- | --- |
|  | Adjusted arithmetic mean, s (SE)^a^ | Adjusted geometric mean, s (SE)^b^ | Median, s | Adjusted arithmetic mean, s (SE)^a^ | Adjusted geometric mean, s (SE)^b^ | Median, s |
| Placebo | 448.1 (16.7) | 369.8 (11.9) | 394 | 405.2 (17.0) | 354.3 (12.1) | 346 |
| Olodaterol 5 µg | 492.0 (16.5) | 421.6 (13.5) | 427 | 469.6 (17.2) | 396.3 (13.7) | 397 |
| Olodaterol 10 µg | 494.2 (16.7) | 420.7 (13.6) | 445 | 444.9 (17.2) | 391.5 (13.6) | 380 |

^a^Calculated based on a mixed effects repeated measures model on original scale; ^b^calculated based on back-transformation of mean estimated with a mixed effects repeated measures model on log_10_-transformed data
SE: standard error

**Table S3** Geometric mean (SE) endurance time after 6 weeks by GOLD (full analysis set)

| Study | GOLD 2 | | | GOLD 3/4 | | |
| --- | --- | --- | --- | --- | --- | --- |
|  | Patients, n | Adjusted geometric mean (SE) endurance time, s | Improvement compared to placebo, % | Patients, n | Adjusted geometric mean (SE) endurance time, s | Improvement compared to placebo, % |
| 1222.37  Placebo  Olodaterol 5 µg   Olodaterol 10 µg | 91 94 93 | 410.5 (24.3) 455.9 (26.8) 462.3 (27.3) | 11.1 12.6 | 45  46 43 | 288.7 (24.3) 348.2 (29.1) 335.2 (28.5) | 20.6 16.1 |
| 1222.38  Placebo  Olodaterol 5 µg   Olodaterol 10 µg | 105 103 102 | 372.7 (18.7) 412.2 (20.8) 412.3 (20.9) | 10.6 10.6 | 40 37 37 | 305.5 (24.7) 350.0 (29.0) 338.2 (28.0) | 14.6 10.7 |

GOLD: Global initiative for chronic Obstructive Lung Disease; SE: standard error

**Table S4** Peak expiratory flow outcomes at 6 weeks (full analysis set)

| Treatment | Planned time, min | Adjusted mean (SE) difference from placebo after 6 weeks, L/s |
| --- | --- | --- |
| Study 1222.37  Olodaterol 5 µg  Olodaterol 10 µg  Olodaterol 5 µg  Olodaterol 10 µg | -0.30 (pre-dose/trough)  60 (post-dose) | 0.32 (0.06)^a^ 0.30 (0.06)^a^ 0.59 (0.06)^a^ 0.62 (0.06)^a^ |
| Study 1222.38  Olodaterol 5 µg  Olodaterol 10 µg  Olodaterol 5 µg  Olodaterol 10 µg | -0.30 (pre-dose/trough)  60 (post-dose) | 0.28 (0.06)^c^ 0.29 (0.06)^d^ 0.58 (0.07)^c^ 0.55 (0.07)^d^ |

^a^n = 137; ^b^n = 136; ^c^n = 143; ^d^n = 139
*p* < 0.0001 for all
SE: standard error

**Fig. S1** Hierarchical testing order: each test was considered confirmatory only if all of the previous tests were positive


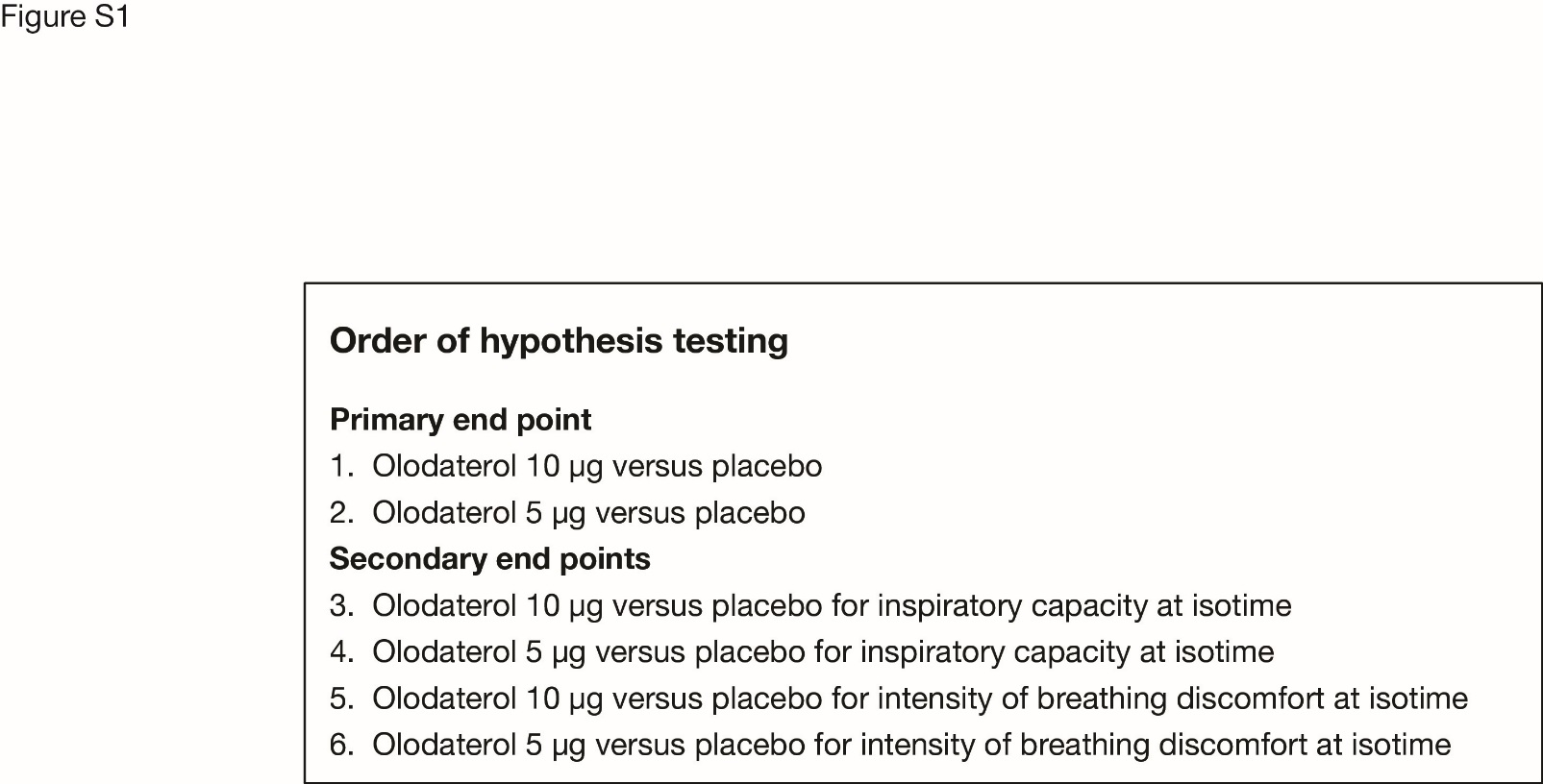


**Fig. S2** Participant flow in (a) Study 1222.37 and (b) Study 1222.38
QD: once daily


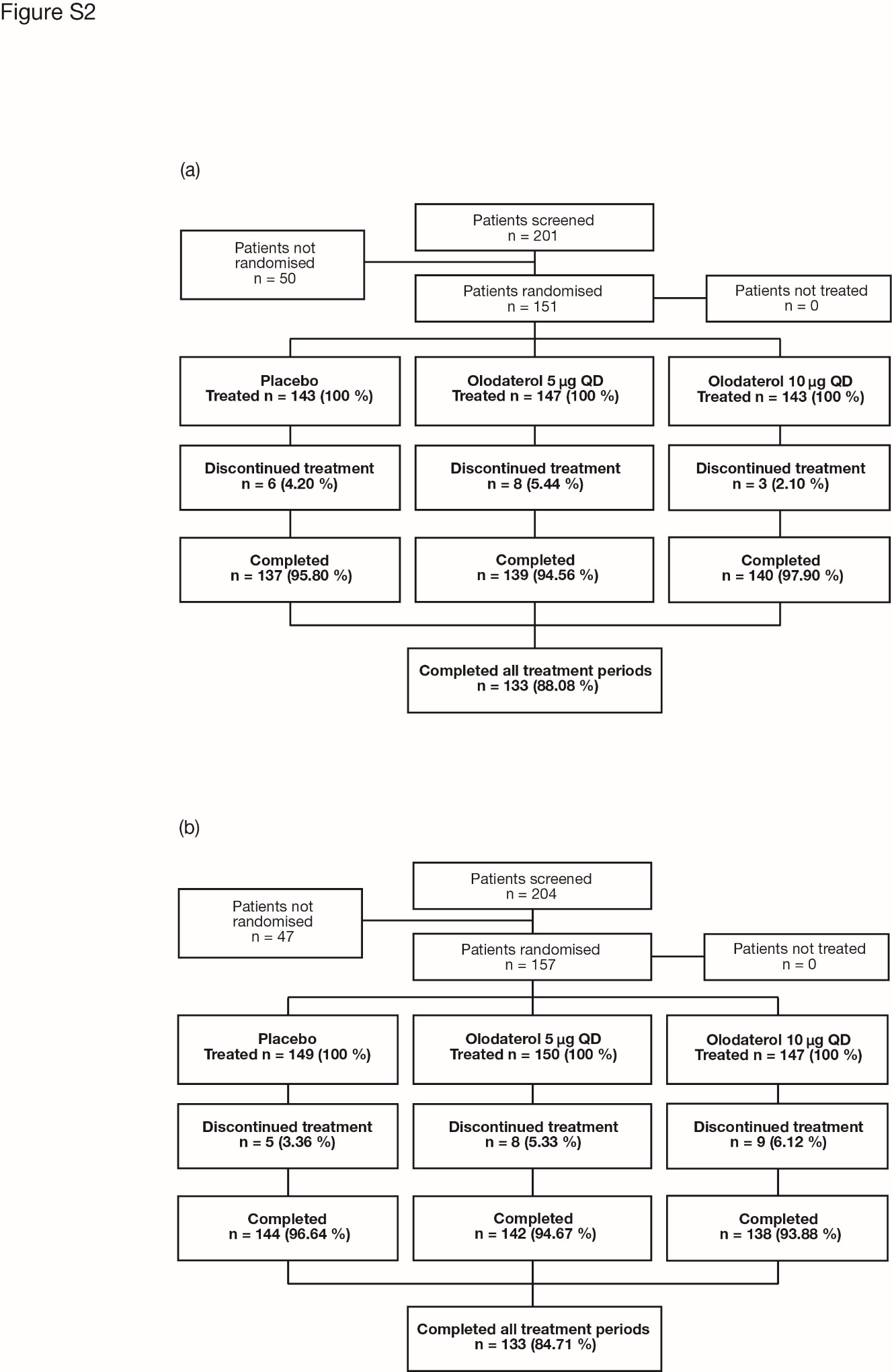


**Fig. S3.** Exercise endurance time after 6 weeks for (a) Study 1222.37 and (c) Study 1222.38. Log_10_-transformation of exercise endurance time after 6 weeks for (b) Study 1222.37 and (d) Study 1222.38 (full analysis set)


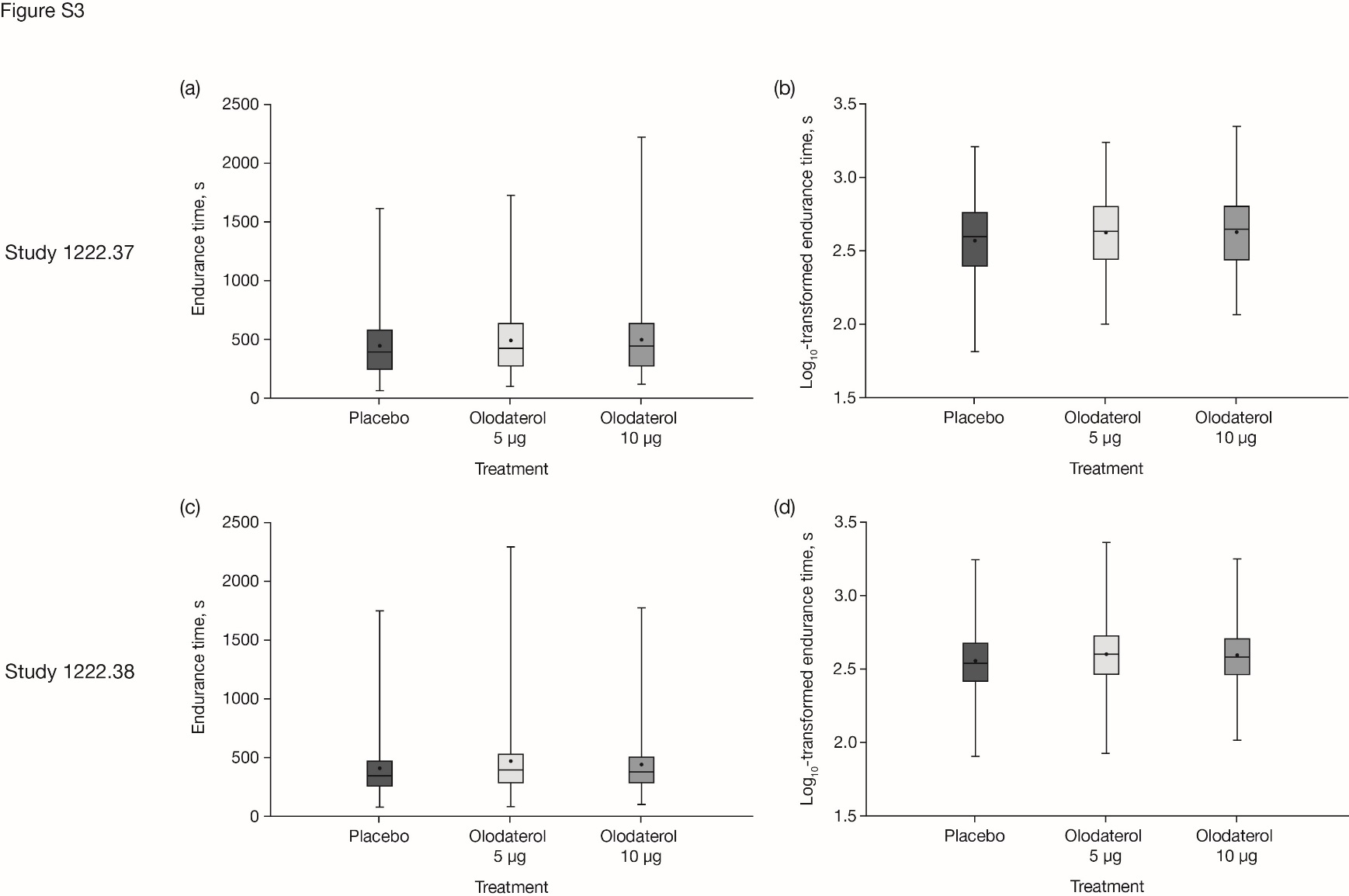

Supplement: Additional file 1: — Table S1. Pulmonary medication use prior to enrolment (treated set). Table S2. Arithmetic mean, geometric mean and median endurance times after 6 weeks (full analysis set). Table S3. Geometric mean (SE) endurance time after 6 weeks by GOLD (full analysis set). Table S4. Peak expiratory flow outcomes at 6 weeks (full analysis set). Figure S1. Hierarchical testing order: each test was considered confirmatory only if all of the previous tests were positive. Figure S2. Participant flow in (a) Study 1222.37 and (b) Study 1222.38. Figure S3. Exercise endurance time after 6 weeks for (a) Study 1222.37 and (c) Study 1222.38. Log10-transformation of exercise endurance time after 6 weeks for (b) Study 1222.37 and (d) Study 1222.38 (full analysis set). (DOCX 631 kb) [file 12931_2016_389_MOESM1_ESM.docx]
